# Characterising the Profile of Everyday Executive Functioning and Relation to IQ in Adults with Williams Syndrome: Is the BRIEF Adult Version a Valid Rating Scale?

**DOI:** 10.1371/journal.pone.0137628

**Published:** 2015-09-10

**Authors:** Darren R. Hocking, Jessica Reeve, Melanie A. Porter

**Affiliations:** 1 Olga Tennison Autism Research Centre, School of Psychology and Public Health, La Trobe University, Melbourne, Victoria, Australia; 2 Psychology Department, Macquarie University, Sydney, New South Wales, Australia; 3 ARC Centre of Excellence in Cognition and its Disorders, Macquarie University, Sydney, New South Wales, Australia; University of Leuven, BELGIUM

## Abstract

Although there is evidence of a distinct profile of executive dysfunction in Williams syndrome (WS), a rare genetically based neurodevelopmental disorder, the utility of informant reports of everyday executive function (EF) impairments and their relation to intelligence is not yet clear. Here we aimed to evaluate the functional impact of executive dysfunction in adults with WS and to establish the validity of child and adult versions of the most commonly used rating scale for EF assessment, the Behaviour Rating Inventory of Executive Function (BRIEF). We were also interested in whether distinct components of everyday EF relate to intelligence in WS. Parent report child (BRIEF-C) and adult (BRIEF-A) ratings were collected on 20 adults with WS (aged 18.5 to 53 years), with a mean IQ of 60.95 (*SD =* 17.67). Neuropsychological measures of EF included: The Shape School Test (Espy, 2007); select subdomains of EF from the Woodcock-Johnson III Tests of Cognitive Abilities, Australian Adaptation (WJ III COG); and select subdomains from the Vineland Adaptive Behaviour Scales, Second Edition—Parent Survey (Vineland-II). Results showed that the BRIEF-A, but not the BRIEF-C, was the most highly correlated with neuropsychological measures of EF, suggesting that it was a valid measure of the profile of EF impairments in adults with WS. The profile of everyday EF dysfunction revealed relative impairments in monitoring, working memory, planning and organisation in WS. In addition, both neuropsychological and rating scale measures showed an association between the *shifting* component of EF and intelligence. These findings indicate that the BRIEF-A is a valid measure of the multidimensional nature of real-world impairments in EF, and highlight its utility as a less labor intensive and low-cost screening tool for measuring specific EF impairments that could become the focus of targeted intervention in adults with WS.

## Introduction

It is often the case that clinicians or researchers need to utilise rating scales and questionnaires as a practical way of quantifying cognitive or psychological functioning in both typical and atypical populations. Although traditional neuropsychological measures arguably provide objective and standardised cognitive assessments, the use of self-report or parent-report questionnaires can provide a more multifaceted measure of everyday behaviours in real life contexts [1]. However, in atypically developing populations with intellectual disability (ID), the extent to which reliability and validity is affected based on the developmental appropriateness of a particular rating scale, and its application to a specific neurodevelopmental disorder, is an important yet neglected issue in developmental neuropsychology.

One domain of cognitive functioning that currently has no universally accepted method of assessment in neurodevelopmental disorders is executive function (EF) skills. EF is an umbrella term for a set of higher-order cognitive processes that organise and regulate goal-directed behaviour in novel and challenging situations [2–4]. EF includes complex cognitive functions such as: cognitive flexibility; task initiation and response inhibition; planning and organisation; working memory and regulation of emotion and behaviour [5–7]. Even within each aspect of EF, such as working memory, there are often presumed to be dissociable components. For example, one influential theory of working memory is Baddeley’s model [8,9], which identifies two domain-specific temporary storage systems for verbal (“phonological loop”) and visuospatial (“visuospatial sketchpad”) information, and a limited-capacity central executive involved in the control and regulation of cognitive processes. It is well established that the central executive is further fractionated into three separable but not completely independent component processes, namely: the ability to shift between mental sets or tasks (‘shifting’); selectively attending to stimuli and inhibiting prepotent responses (‘inhibition’); and updating and monitoring in working memory (‘updating’) [10]. There has been considerable controversy as to whether these components of EF can be viewed as separable or rather as a unitary construct in early development [11–13], and this controversy equally applies to neurodevelopmental disorders associated with ID.

Willner et al. [14] employed child versions of performance-based measures of EF in adults with mild to moderate IDs, and found three factors (i.e. inhibition, shifting and updating working memory) that resembled the tripartite model of EF proposed in typical development [10]. Thus, there appears to be some utility in adopting this multi-dimensional model of EF in neurodevelopmental disorders where it is possible that some components of EF may be more impaired than others relative to overall mental age (MA) level. This has important implications for targeted interventions in neurodevelopmental disorders such as autism where select deficits in behavioural flexibility or shifting have been observed [15–17]. For example, preliminary studies in autism provide support for improvements in core behaviour regulation problems (i.e. ability to follow rules, make transitions and be flexible) when behavioural flexibility is directly targeted in contextually-based EF interventions [18].

Williams syndrome (WS) is a rare (1 in 7,500) neurodevelopmental disorder resulting from a microdeletion of approximately 25–28 genes on the long arm of chromosome 7q11.23 [19,20]. The syndrome is characterised by distinctive facial morphology, cardiovascular problems, mild to moderate intellectual disability, and gregarious and empathic social personalities. Individuals with WS have a high frequency of attention-deficit/hyperactivity disorder (ADHD) and/or Generalised anxiety disorder as co-morbid diagnoses, with a higher than expected prevalence compared to the general population in both instances [21,22].

The disorder has attracted a great deal of interest from developmental neuropsychologists due to a unique profile of strengths and weaknesses in the neuropsychological and EF profile. These include a relative proficiency in some aspects of language and short-term verbal memory, alongside weaknesses in non-verbal domains associated with visuospatial and visuomotor abilities [23–25]. Recent studies have generally been consistent with a fractionation of EFs that are more or less compromised in individuals with WS [26–29]. There are an increasing number of studies that suggest poor performance in WS relative to both MA matched typically developing (TD) individuals and task norms in: response inhibition [30–32]; dual-task coordination [33,34]; shifting, working memory, planning and organisation [27]. These deficits across a number of EF domains appear to be more specific to the visuospatial modality in WS when compared to the verbal domain [27,31]. With respect to working memory (WM), previous studies have indicated that individuals with WS have relatively weaker visuospatial WM compared to MA, whereas their performance on verbal WM tasks is comparable to MA matched TD children [35]. This has been suggested as a syndrome-specific profile in a recent study by Carney et al. [36], who examined the effect of task modality on executive function in WS and Down syndrome (DS) when compared to TD children. Although the typical profile of weaker visuospatial WM was indicated in the WS group, there were no developmental delays in verbal short-term memory between individuals with WS and TD children matched for MA or chronological age. These findings suggest that EF is not uniformly impaired in individuals with WS.

In addition to limitations of face-to-face performance measures of EF in WS and other neurodevelopmental disorders, there is considerable controversy regarding the precise relationships between IQ and performance-based EF skills. Friedman et al. [37] examined performance on three types of EF (inhibiting, working memory and shifting) in a large sample of typically developing adolescents aged 16 to 18 years, and found working memory to be highly correlated with IQ measures, but not shifting or inhibitory control. However, in children with specific learning and intellectual disabilities, evidence is consistent with the notion that working memory is also associated with specific learning disabilities after taking into account variations in IQ [38]. When comparing children with mild learning disabilities to a group with more severe intellectual disability (normal vs low IQ), Maehler and Schuchardt [38] revealed no significant differences in working memory between the two groups of children, despite their varying levels of intelligence. To further add to these conflicting findings, Osório et al. [3] recently showed that working memory (but not shifting or inhibition) was most strongly associated with IQ in both individuals with WS and in typically developing (TD) controls, albeit that the strength of the association was considerably higher in the WS group. Notably, the association among the three EFs was substantially greater in individuals with WS than in the TD group, suggestive of a global compromise of EFs in WS [3]. However, the relationships between the everyday profile of EF skills and intelligence are, as yet, unclear in WS, and are therefore worthy of further investigation.

In addition to the theoretical debate regarding the multi-dimensional nature of EF in neurodevelopmental disorders, there are several inherent limitations of performance-based measures of EF in neurodevelopmental disorders. One particular issue is that many performance-based tests of EF rely heavily on lower level abilities that are typically impaired in individuals with compromised intellectual functions, such as processing speed and fine motor control. Another important limitation is that relating to the structured and interactive nature of performance-based tests of EF that may limit their ecological validity in terms of measuring the control of everyday real-world behaviour. That is, standardised one-on-one testing environments of performance-based tests of EF provide structure and cues, and monitoring that may reduce cognitive demands associated with critical EF processes that otherwise would be impaired in less structured settings [1]. Thus, performance-based measures may not be representative of more complex everyday EF in the real-world environment. These limitations can be overcome with the development of rating scale approaches that measure EF demands in the everyday environment (e.g. the BADS-C, [39]), and parent-report behaviour rating scales such as the Behaviour Rating Inventory of Executive Function for children (BRIEF-C, [40]), which can complement face-to-face performance-based measures. Importantly, a related issue is whether rating scales developed for children can be considered developmentally appropriate when employing them for adults with ID, especially when overall MA level would be more commensurate with the use of a child version of a rating scale. This is a particularly relevant issue for studies using rating scales in WS that typically include both children and adults across a wide age range [27,41], and where child versions of rating scales are often utilised, even for adults with WS.

The Behavior Rating Inventory of Executive Function—Adult version (BRIEF-A) was originally developed and adapted for use with typically developing adults, but has since been utilised for a range of acquired and neurodevelopmental disorders including: autism [42–44][; attention deficit hyperactivity disorder (ADHD; Rotenberg-Shpigelman et al., 2008), and individuals with Traumatic Brain Injury (TBI, [45]). The adult version of the BRIEF assesses functional problems related to everyday executive functioning and is separated into two indices (Metacognition and Behavioural Regulation), and nine scale scores. The Metacognitive Index (MI) consists of five scales: task monitor; organisation of materials; plan/organise; working memory and behavioural initiation. The Behavioural Regulation Index (BRI) contains four scales: emotional control; shift; inhibit and self-monitor. Although the psychometric properties of the child BRIEF have been well documented [46,47], there is very little information on the validity of the BRIEF-A version in adult samples with neurodevelopmental disorders associated with ID. The existing studies that have examined EF profiles using the BRIEF in young children with ID are very limited. Lee et al. [48] examined EF profiles with the preschool version of the BRIEF in young children with Down syndrome (DS) and found elevations on the global EF composite, as well as significant deficits in working memory and planning beyond those expected for overall developmental level (see also [49]). Given that very little is known about how EF skills may change in adulthood in people with WS, it is crucial that further studies examine the functional impact of EF deficits in adulthood using validated measures of everyday EFs that are developmentally appropriate for the specific age group under investigation.

### Current study

The primary aim of this study was to examine the functional impact of executive dysfunction in an adult sample of WS individuals to establish the most valid version of the BRIEF. In order to achieve this aim, we explored the following research questions. The first question was whether parent ratings differed depending on whether they used the child or adult version of the BRIEF. This was examined through the utilisation of the BRIEF-C and the BRIEF-A questionnaires in adults with WS. The second question related to whether the BRIEF-C or the BRIEF-A was most related to performance-based measures of EF. Finally, once the more valid version of the BRIEF had been established, the third research question related to the extent to which the BRIEF questionnaire captured the multi-dimensional nature of EF, and the possible interrelationships between EF components and IQ in adults with WS.

Based on the lack of previous research in this area, no specific hypotheses were made with respect to the first two research questions. However, with regard to the third research question, and consistent with previous findings [3,26,27], it was hypothesised that adults with WS would display elevated levels of executive dysfunction in inhibition, planning, and working memory (H_1_). In relation to the extent to which BRIEF ratings are related to IQ, Osório and colleagues’ [3] study reported both working memory and inhibiting to be associated with IQ, but working memory was found to explain a greater proportion of variance in IQ in the WS group. Therefore, it was hypothesised that while both working memory and inhibition would be significantly correlated with IQ, it was predicted that there would be a stronger association between the EF component of working memory and IQ in adults with WS (H_2_).

## Method

### Participants

Twenty adults with a diagnosis of WS (11 males and 9 females) and their parents were recruited through the Williams Syndrome Family Support Group (Victoria) and the Williams Syndrome Association Australia. All participants with WS had their diagnosis confirmed with the positive fluorescent in situ hybridisation (FISH) test and displayed the typical ~1.6 Mb heterozygous microdeletion at 7q11.23 [50]. Participants were screened for a history of psychological, developmental or neurological impairments unrelated to the syndrome, such as ADHD, epilepsy or traumatic brain injury assessed by an experienced clinical neuropsychologist (last author). No participant was excluded from the study based on these criteria, as no potential participant displayed the above. The participants’ chronological age ranged from 18.5 to 53 years (*M =* 27.74, *SD =* 8.53). The mental age and IQ for each participant was assessed using the Woodcock-Johnson III Tests of Cognitive Abilities, Australian Adaptation (WJ III COG; [51]). Four participants were unable to complete neuropsychological testing at the time of their participation in the current study due to illness or misadventure. An IQ test was conducted (the Woodcock-Johnson Tests of Cognitive Abilities–Revised, Australian Adaptation or WJ-R COG) approximately three to five years prior with each of these individuals as part of another study. Mental age for these four participants ranged from 5.75 to 8.08 years (*M =* 6.61, *SD =* 1.06) and overall IQ ranged from 50 to 64 (*M =* 53, *SD =* 7.72). These four participants were, therefore, representative of the larger study cohort. Mental age ranged from 5 to 10.83 years (*M =* 7.15, *SD =* 1.83). Participants performed in the mild to moderate range of intellectual disability, on average, with IQs ranging from 25 (severe) to 86 (low average) (*M =* 60.84, *SD =* 18.15). The mean and range of IQs in the current sample was highly consistent with that reported for intelligence in WS (Mervis et al., 2000), and with previous studies of cognitive heterogeneity in WS (Martens et al., 2008; Porter & Coltheart, 2005). Due to low reading ability and IQ, all participants with WS provided verbal consent after a brief description of the study, and parents provided signed “surrogate” consent according the declaration of Helinski. Ethics approval for the consent procedure and this study was gained from the Macquarie University Human Research Ethics Committee (reference number: 5200900071).

### Materials

#### The Child Behaviour Rating Inventory of Executive Functioning (Parent Form)—BRIEF-C

The BRIEF-C is designed to assess the executive function behaviours of children aged from 5 to 18 years in a home and school environment [40]. The questionnaire consists of 86 standardised items that measure different aspects of executive function. Seventy-two of those items fall within eight clinical scales (Inhibit, Shift, Emotional Control, Initiate, Working Memory, Plan/Organise, Organisation of Materials, and Monitor). These scales combine to form two indexes (Behavior Regulation Index [BRI] and the Metacognition Index [MI]) and one composite summary score (Global Executive Composite [GEC]). The structure of the BRIEF-C rating scale is outlined in S1 Table. For each clinical scale and index, a *T* score can be derived with higher scores indicating greater degrees of executive dysfunction, with scores at or above 65 suggesting clinical significance [40]. Psychometric properties show high internal consistency with Cronbach alpha coefficients ranging from .80 to .98 and good test-retest reliability ranging from .76 to .85 [40].

#### Behaviour Rating Inventory of Executive Function—Adult Version (Parent Report Form)—BRIEF-A

The BRIEF-A is designed to assess the everyday executive functioning behaviours of adults aged from 18 to 90 years [52]. The questionnaire consists of 75 standardised items, of which items measure different aspects of executive function within nine clinical scales (Inhibit, Shift, Emotional Control, Self-Monitor, Initiate, Working Memory, Plan/Organise, Organisation of Materials, and Task Monitor). The nine clinical scales combine to form two indices (BRI and the MI) and one composite summary score (GEC). The structure of the BRIEF-A rating scale is outlined in S2 Table. For each scale, *T* scores can be derived with higher scores reflecting greater degree of executive dysfunction and levels of impairment, with scores at or above 65 suggestive of clinical significance. Internal consistency is high with alpha coefficients ranging from .80 to .98, and high test-retest reliability ranging from .91 to .94 [52].

#### The Shape School test

Inhibitory control was assessed using the Shape School test [53]. Four conditions (control, inhibit, switch, and both) were presented in the same fixed order from easiest to most difficult conditions as per recommendations [53]. The participant is shown rows of coloured squares and circles, which are depicted as pupils in “The Shape School”. The first condition required each participant to name the colour of each pupil (Control Condition). The second condition required participants to inhibit salient yet irrelevant information and only name the colours of pupils with a happy facial expression while ignoring the sad pupils (Inhibit Condition). In the third condition, some pupils are drawn with hats and the participants are required to switch between naming the hatted pupil’s colour and the hatless pupil’s shape (Switch Condition). The final condition consists of both the happy/sad faced pupils and the hat/hatless pupils (Both Condition). The participant is required to name the shape of the happy pupil with the hat, and name the colour of the happy pupil without a hat while refraining from naming the sad pupil (hatted or hatless). Responses and the time taken to complete each of the conditions were recorded. An efficiency score was calculated from the naming speed and accuracy scores as per recommendations [53].

#### The Vineland Adaptive Behaviour Scales, Second Edition (Parent Survey)—Vineland-II

The Vineland-II is a standardised semi-structured parent interview that measures adaptive functioning [54]. The Vineland-II consists of 11 subdomains grouped into four domain composites (Communication, Daily Living Skills, Socialisation, and Motor Skills), with the domain composites used to derive the adaptive behaviour composite. In addition, the Vineland-II provides a Maladaptive Behaviour Index (comprising three subscales; Internalising, Externalising, and Other) that measures undesirable behaviours that may inhibit the development of an individual’s adaptive functioning. Raw scores were converted into standard scores (with population *M* = 100, *SD* = 15) based on chronological age. Lower scores reflect greater maladaptive behaviour with scores 2 standard deviations below the normative mean (score of 69) indicating low level of adaptive functioning. The Vineland-II has excellent levels of reliability and internal consistency [54].

#### Woodcock-Johnson III Tests of Cognitive Abilities, Australian Adaptation—WJ III COG

The WJ III COG is a standardised measure of intelligence designed for individuals between the ages of 2 and 90 years. The WJ III COG is based on the Cattell-Horn-Carroll (CHC) theory proposing two types of intelligence—fluid (*Gƒ*) and crystallised (*Gc*) intelligence, or innate or learned intelligence [55]. The WJ III COG can be explored at three levels (known as Stratum I, II, and III) [55]. Stratum I consists of 20 individual tests, each measuring many of the specific abilities of Gf and Gc intelligence. Further, the battery provides a measurement of seven Stratum II (CHC) factors that are derived from two qualitatively different Stratum I abilities. In addition to Stratum II, several clinically useful clusters may also be obtained by combining several of the Stratum I abilities. Finally, the WJ III COG yields an overall (Stratum III) General Intellectual Ability (GIA) or single *g* factor, which is similar to the Full Scale IQ (FSIQ) of the Wechsler Scale of Intelligence. In accordance with the WJ III COG manual [55], executive functioning is measured through the Broad Attention, Working Memory, and Executive Process Clinical Clusters.

The raw scores on the WJ III can be converted into standard scores (with population *M* = 100, *SD* = 15) based on the age of the participant, with lower scores reflecting greater cognitive difficulties (a score of 69 or below is classified as “very low”) (Mather & Woodcock, 2001). WJ III COG is psychometrically sound with reliability coefficients above .80. Median reliabilities for each cluster are typically at .90 or higher (McGrew & Woodcock, 2001). The GIA measures also display high reliability, with the GIA-Ext ranging from .98 to .99 [55].

### Data analyses

To compare ratings on BRIEF-C and BRIEF-A clinical scales, indices and GEC scores, pairwise comparisons and cross-tabulations were employed. In addition, Cohen’s kappa coefficient [56] was reported to provide a measure of agreement, corrected for chance agreement, between the scores on the BRIEF-C and BRIEF-A (0 = poor agreement, .20 –slight agreement, 0.4 = fair agreement, 0.6 = moderate agreement, 0.8 substantial agreement, 1.0 = almost perfect agreement). *T* scores were utilised in the analyses of the BRIEF-C and BRIEF-A measures, and were calculated based on chronological age. Item analysis was then conducted to compare each item pair on the BRIEF-C and BRIEF-A rating scales in order to investigate how similar each item pair was rated. Identical items were chosen for analysis as these were expected to be highly correlated. Identical items were also investigated using pairwise comparisons and Cohen’s kappa coefficient. Overall, the BRIEF-C Inhibit (*D*(20) = .20, *p* = .033) and Shift (*D*(20) = .23, *p* = .009) clinical scales and all identical items violated the assumption of normality, and hence nonparametric tests were employed for these scales.

Due to the small sample size, and in order to minimise the likelihood of a Type-II error the *p* value was set to 0.05 for all analyses (see [57]). For any coefficients at or near the .05 level of significance, moderate to large effect sizes would rule out the likelihood of a Type-I error [57], and, as such, effect sizes were reported for all analyses (0.2 = small effect size, 0.5 = medium effect size, 0.8 = large effect size) [58]. To examine the profile of EF deficits on the BRIEF, repeated measures analyses of variance (ANOVA) was carried out to compare the performance of the adults with WS on each of the BRIEF-A clinical scales, indices and GEC scores. Pearson correlation coefficients were used to investigate whether BRIEF-A ratings were associated with IQ.

## Results

### Descriptive analysis

Table 1 displays descriptive statistics for the clinical scales, indices and GEC scores of the BRIEF-C and BRIEF-A *T* scores. The BRIEF-C revealed four clinical scales to be in the clinically significant range (≥65), namely Initiate, Working Memory, Plan/Organise, and Monitor, as well as the MI and GEC indexes. The BRI and its corresponding scales were all found to be within normal limits. The BRIEF-A revealed five clinical scales to be clinically significant, namely Shift, Initiate, Working Memory, Plan/Organise, and Task Monitor, as well as the MI and GEC indexes.

**Table 1:** Descriptive statistics for adult WS on the BRIEF-C and BRIEF-A T scores (N = 20).

BRIEF Clinical Scales, Indices and GEC	BRIEF-C		BRIEF-A	
	*M*	*SD*	*M*	*SD*
**BRI**	**59.80 (43–80)**	**12.34**	**59.60 (40–79)**	**11.16**
Inhibit	57.45 (42–81)	11.66	58.05 (39–76)	10.99
Shift	62.10 (47–87)	10.89	65.00 (42–84)	11.24
Emotional Control	57.00 (37–77)	12.52	56.85 (39–77)	12.05
Self-Monitor* ^a^	-	-	57.15 (37–75)	11.19
**MI**	**70.75 (52–88)**	**10.99**	**69.70 (46–83)**	**10.18**
Initiate	66.15 (43–83)	10.82	65.05 (48–87)	9.70
Working Memory	72.00 (50–93)	12.32	69.55 (41–86)	13.17
Plan/Organise	70.15 (45–89)	12.11	68.70 (43–82)	10.46
Org. of Materials*	60.15 (46–72)	7.91	63.15 (45–78)	9.59
Monitor* ^a^	67.95 (51–88)	11.81	-	-
Task-Monitor	-	-	70.3 (45–87)	12.19
**GEC**	**67.70 (49–85)**	**10.58**	**65.8 (43–83)**	**10.76**

*Note*. The minimum and maximum *T* scores of WS participants from the BRIEF-C and BRIEF-A of each clinical scale, index and GEC are in parentheses.

* Indicates significantly different clinical scale, index, and GEC *T* score on the BRIEF-C or BRIEF-A.

^a^ Significant paired t-tests between the BRIEF-C Monitor and BRIEF-A Self-Monitor scale *T* scores.

### Item analyses and reliability for BRIEF-C and BRIEF-A

To examine the differences between each of the clinical scales, indices and GEC scores across the BRIEF-C and BRIEF-A, paired samples *t*-tests were performed. Overall, parents rated their son or daughter with WS as significantly higher on the BRIEF-C Monitor scale than on the BRIEF-A Self-Monitor scale (*t*(19) = 4.61, *p* < .001, *r* = .73). Since the BRIEF-C Inhibit and Shift scales were not normally distributed, the Wilcoxon signed-rank test was performed, but no other significant differences emerged (see Table 1).

The ratings of the identically worded items were analysed in order to determine whether the scores of the BRIEF-C and BRIEF-A questionnaires are interpreted by parents in the same way given the context of the adult and child questionnaire and the difference in the surrounding items (Table 2). Paired samples *t*-tests revealed no significant differences in parent ratings on the nine identical item pairs. Cohen κ statistics for interrater agreement for parent ratings between the BRIEF-C and BRIEF-A identical item pairs can be seen in Table 2. The strength of agreement ranges from poor to moderate, indicating that parents showed different ratings across the identical items on the BRIEF-C and BRIEF-A. The lowest Cohen κ statistic (indicating a poor strength of agreement) can be seen on the item “Has good ideas but cannot get them on paper”. The highest Cohen κ statistic is seen for the item “Is impulsive”, indicating a moderate agreement between the identical pair.

**Table 2:** Measurement of agreement between the identical items on the BRIEF-C and the BRIEF-A.

	BRIEF-C		BRIEF-A		
Item	Clinical Scale	Item Number	Clinical Scale	Item Number	κ
1. “Talks at the wrong time”	Inhibit	65	Self Monitor	23	.32*
2. “Overreacts to small problems”	Emotional Control	1	Emotional Control	33	.42**
3. “Mood changes frequently”	Emotional Control	26	Emotional Control	69	.33*
4 “Has a short attention span”	Working Memory	9	Working Memory	35	.54**
5. “Has good ideas but cannot get them on paper”	Plan/Organise	15	Plan/Organise	47	-.07
6. “Has a messy closet”	Org. of Materials	72	Org. of Materials	7	.35*
7. “Does not check work for mistakes”	Monitor	14	Task Monitor	18	.40*
8. “Makes careless mistakes”	Monitor	21	Task Monitor	41	.59**
9. “Is impulsive”	-	82^a^	Inhibit	73	.60**

*Note*.

κ = Cohen’s kappa coefficient measuring the agreement between the BRIEF-C and BRIEF-A identical item pair ratings. Items have been reproduced from “Behavior Rating Inventory of Executive Function” by G. A. Gioia, P. K. Isquith, S. Guy, and L. Kenworthy, 2000a, p. 38–41. Copyright 2000 by the Psychological Assessment Resources, Inc. Also adapted from the “Behaviour Rating Inventory of Executive Function-Adult Version” by R. M. Roth, P. K. Isquith, and G. A. Gioia, G. A., 2005, p. 49–51. Copyright 2005 by the Psychological Assessment Resources, Inc.

^a^ Item 82 from the BRIEF-C does not load onto a clinical scale.

** *p* < 0.01

* *p* < 0.05.

### Validity of BRIEF-C and BRIEF-A rating scales in measuring EF in WS

#### Relationship between the Shape School, WJ III COG, Vineland-II, and BRIEF-C

No significant relationships were identified between the performance-based measures and the BRIEF-C clinical scales, indices, and GEC scores (*p* > .05) (see S3, S4, S5 Tables).

#### Correlational analyses for Shape School and BRIEF-A

Correlations were examined between the Shape School and the BRIEF-A using Pearson’s correlation coefficients. There was a significant negative correlation between the Shape School Switch condition and the BRIEF-A Shift clinical scale (r = -.47, *p* = .042) (Table 3). In addition, the Shape School Both condition was significantly negatively related to Shift (r = -.60, *p* = .006), Working Memory (r = -.51, *p* = .026), Plan/Organise (r = -.64, *p* = .003), MI (r = -.59, *p* = .009), and the GEC (r = -.49, *p* = .034). There were no other significant relationships between the Shape School conditions and BRIEF-A scales and indices (*p* > .05).

**Table 3:** Correlations Between the Shape School and the BRIEF-A (T Scores).

	Shape School Conditions		
BRIEF-A Clinical Scales, Indices, and GEC	Inhibit (n = 19)	Switch (n = 19)	Both (n = 19)
**BRI**	**-.27**	**-.24**	**-.34**
Inhibit	-.09	.02	-.08
Shift	-.25	-.47*	-.60**
Emotional Control	-.32	-.22	-.22
Self-Monitor	-.22	-.12	-.31
**MI**	**-.37**	**-.25**	**-.59** **
Initiate	-.35	-.10	-.45
Working Memory	-.27	-.35	-.51*
Plan/Organise	-.42	-.34	-.64**
Task-Monitor	-.07	-.15	-.42
Org. of Materials	-.23	-.02	-.22
**GEC**	**-.33**	**-.27**	**-.49** *

*Note*. Scores represent Pearson’s correlation coefficient.

** *p* < 0.01.

* *p* < 0.05.

#### Correlational analyses for WJ III COG and BRIEF-A

Correlations were explored between the WJ III COG measures of EF and the BRIEF-A using Pearson’s correlation coefficients (Table 4). The WJ III COG Broad Attention clinical cluster was significantly and negatively correlated with the BRIEF-A clinical scales Shift (*r* = -.64, *p* = .007), Working Memory (*r* = -.52, *p* = .038), and Plan/Organise (*r* = -.55, *p* = .026). The Executive Processes clinical cluster was significantly and negatively related with BRIEF-A Shift (*r* = -.71, *p* = .003), Working Memory (*r* = -.54, *p* = .039), and Plan/Organise (*r* = -.64, *p* = .010) scales. There were no other significant relationships between the WJ III COG tests of EF and the BRIEF-A clinical scales and indices (*p* > .05).

**Table 4:** Correlations Between the WJ III COG and the BRIEF-A (T Scores).

	WJ III COG Clinical Clusters		
BRIEF-A Clinical Scales, Indices, and GEC	Working Memory (n = 15)	Broad Attention (n = 16)	Executive Processes (n = 15)
**BRI**	**-.20**	**-.38**	**-.43**
Inhibit	-.09	-.34	-.25
Shift	-.51	-.64**	-.71**
Emotional Control	-.16	-.33	-.39
Self-Monitor	-.09	-.18	-.32
**MI**	**-.05**	**-.33**	**-.44**
Initiate	-.05	-.28	-.39
Working Memory	-.35	-.52*	-.54*
Plan/Organise	-.37	-.55*	-.64*
Task-Monitor	-.10	-.36	-.41
Org. of Materials	.23	-.03	-.15
**GEC**	**-.21**	**-.44**	**-.51**

*Note*. Scores represent Pearson’s correlation coefficient.

** *p* < 0.01.

* *p* < 0.05.

#### Correlational analyses for Vineland-II and BRIEF-A

Correlations between the Vineland-II adaptive behaviour domains and the BRIEF-A revealed a significant positive correlation between Vineland-II Externalising Behaviours and BRIEF-A Emotional Control (*r* = .54, *p* = .039) (Table 5).

**Table 5:** Correlations Between the Vineland-II and the BRIEF-A (T Scores).

	Vineland-II Domains				
			Maladaptive Behaviour Subscales		
BRIEF-A Clinical Scales, Indices, and GEC	Adaptive Behaviour Composite (n = 16)	Socialisation Domain (n = 16)	Maladaptive Behaviour Index (n = 15)	Internalising Behaviours (n = 15)	Externalising Behaviours (n = 15)
**BRI**	**-.06**	**-.07**	**.12**	**-.15**	**.32**
Inhibit	-.01	-.01	-.12	-.33	-.05
Shift	-.48	-.38	.18	-.04	.13
Emotional Control	.07	.06	.27	.01	.54*
Self-Monitor	.18	.07	-.11	-.31	.21
**MI**	**-.26**	**-.21**	**-.05**	**-.33**	**-.06**
Initiate	-.27	-.23	-.02	-.30	-.00
Working Memory	-.22	-.22	-.18	-.41	-.31
Plan/Organise	-.12	-.14	.11	-.16	.16
Task Monitor	-.42	-.38	-.17	-.39	-.23
Org. of Materials	-.12	.06	-.01	-.17	.00
**GEC**	**-.18**	**-.15**	**.03**	**-.26**	**.12**

*Note*. Scores represent Pearson’s correlation coefficient.

* *p* < 0.05.

### Behavioural profile of everyday executive functioning in WS

To examine BRIEF-A score profiles, repeated-measures ANOVA was conducted to ascertain differences between the clinical scales, indices and GEC scores. Mauchly’s test indicated that the assumption of circularity was not satisfied (χ^2^(65) = 180.24, *p* < .001), and therefore degrees of freedom were corrected using the Huynh-Feldt estimate of sphericity (ε = .64). There were significant differences between the BRIEF-A scores (*F*(7.08, 134.55) = 13.05, *p* = < .001, partial η^2^ = .41), with ratings significantly highest for Task-Monitor, Working Memory, Plan/Organise, MI, and GEC. Moreover, 70% of the current sample scored in the clinically significant range (T ≥ 65) on Task-Monitor, 65% on Working Memory, 60% on Shift, 70% on Plan/Organise, 65% on MI, and 55% on GEC; compared to 35% on Inhibit, 60% on Shift, 30% on Emotional Control, 35% on Self Monitor, 55% on Initiate and 25% on the Behavioural Regulation Index. See S6 Table for behavioural profile on the Shape School, WJ III COG and Vineland-II.

### Relation between IQ and the BRIEF-A

The level of associations between BRIEF-A and IQ were explored using Pearson correlation coefficients. The BRIEF-A Shift clinical scale was significantly and negatively correlated with IQ (*r* = -.52, *p* = .021), indicating that lower IQ score is related to poorer EF abilities in the component of shifting (Table 6). No other significant relationships were identified.

**Table 6:** Correlations Between IQ and the BRIEF-A (T Scores).

BRIEF-A Clinical Scales, Indices, and GEC	IQ
**BRI**	**-.30**
Inhibit	-.17
Shift	-.52*
Emotional Control	-.25
Self-Monitor	-.14
**MI**	**-.23**
Initiate	-.20
Working Memory	-.39
Planning/Organising	-.44
Task-Monitor	-.17
Organisation of Materials	-.11
**GEC**	**-.34**

*Note*. Scores represent Pearson’s correlation coefficient.

* *p* < 0.05.

## Discussion

The primary goal of this study was to examine the functional impact of everyday EF impairments in adults with WS using an in-depth analysis of both neuropsychological measures and parent reported EF rating scales: the BRIEF. We compared the validity of parent reports on the BRIEF adult (BRIEF-A) and child (BRIEF-C) rating scales with respect to their relation to neuropsychological measures of EF, and the behavioural profile of everyday EF in adults with WS. This study also explored interrelationships between distinct components of everyday EF and intelligence. The current study is the first to expand on previous performance-based neuropsychological studies in WS by establishing the validity of adult parent reports of everyday EF in adults with WS [3,26,27].

The current findings indicated that BRIEF-A parent ratings were a more valid measure of EF impairments than the BRIEF-C in adults with WS. This was most apparent when examining identical item level ratings between the BRIEF-C and BRIEF-A, with evidence of poor to moderate interrater agreement between the two BRIEF rating scales. There are several possible explanations for the discrepancies in ratings across the BRIEF-C and BRIEF-A in adults with WS. Firstly, it is possible that the context of the surrounding items may influence parent reported ratings for each item on the child and adult BRIEF scales. Secondly, the discrepancies in parent ratings between the BRIEF-C and BRIEF-A might reflect differences in rating scale instructions: that is, the BRIEF-C directs respondents to consider problem behaviours over ‘the past six months’, while the BRIEF-A requires consideration of problem behaviours only ‘over the past month’. Although it is unlikely that EF abilities would change dramatically over time in adults with WS, it remains feasible that the elevated parent reported BRIEF-C scores could be a reflection of the parents’ inclusion of more problem behaviours over a longer period of time, with greater opportunity for poor behaviour exemplars to occur over a six month time period versus a one month time period. Finally, it is conceivable that expectations vary according to the developmental level (child or adult version) in which EF performance is compared by parents relative to typically developing peers. However, this seems unlikely to account for these discrepancies as the parent ratings indicated a greater degree of EF impairments on the BRIEF-C than the BRIEF-A in adults with WS.

The current findings revealed strong associations between individually administered neuropsychological measures of EF and the BRIEF-A scales and, therefore, provide support for the validity of the adult BRIEF in capturing EF impairments reflected in everyday functioning in adults with WS. These findings are important in view of the lack of correlations between performance measures and rating scales on the BRIEF-C in previous studies, leading to debate as to whether rating scales of EF are actually measuring the same underlying construct [59]. However, the current study findings have revealed distinct associations between the putative EF skills measured by individual BRIEF-A clinical scales and performance measures that tap specific components of EF. Performance on the Shape School switch condition (a measure of cognitive flexibility) was negatively associated with ratings on the BRIEF-A Shift, but not Inhibit scale, while the WJ III COG measure of Executive Processes showed negative associations with several BRIEF-A scales including shifting, working memory and planning/organisation. The Shape School is a measure of two main components of EF commonly included in theoretical accounts of executive control [60,61]: the ability to inhibit a prepotent response (response inhibition), and ability to flexibly adapt to changes in the environment (cognitive flexibility). Diamond [11] has argued that the type of mixed switching is perhaps the most difficult because the neural systems operate in a global, diffuse way, making it easier to shift global behaviours than repeatedly invoke different response sets across trials. We conclude that parent reports of everyday shifting as measured by the BRIEF-A appear to tap into these higher demands on cognitive flexibility in a structured context in adults with WS.

Consistent with previous studies from individually administered neuropsychological measures [2,26,27], we found adults with WS exhibited scores in the clinically significant range on several of BRIEF-A subscales. Contrary to previous reports using neuropsychological assessments of EF in WS [3,30,32,35,36], we did not find greater parent reported ratings on the BRIEF-A Inhibit Scale which measures inhibitory control and impulsivity. The discrepancy between inhibitory control deficits between parent report and performance-based neuropsychological findings could reflect the previous samples that included a wide age range including children with WS (e.g. [26,32]). Additionally, we found no specific deficits on the Shape School Inhibit condition in adults with WS, which contrasts with a previous report of poorer response inhibition relative to developmental and IQ level on the same measure in individuals aged 5 to 46 years with WS [32]. Another possible explanation for these discrepancies may relate to inhibitory control impairments known to be associated with attention deficit hyperactivity disorder (ADHD), which is the most common comorbid diagnosis (up to 67.4%) in children with WS [21,22]. While previous studies have typically included children without assessment for ADHD characteristics, in the current study we screened adults with WS and none had an ADHD diagnosis. Thus, the greater difficulties in inhibitory control in previous studies in children with WS [26,32] may be secondary to ADHD characteristics. This interpretation is consistent with a previous study that found a general preservation of inhibition irrespective of the modality in WS individuals without a diagnosis of ADHD [35], and with previous evidence that impulsivity and ADHD characteristics are more common in children with WS [62].

The results of the current study did not support our hypothesis that working memory would be most strongly associated with intelligence in adults with WS. We expected that previous evidence of a link between working memory and IQ in both WS and typically developing children [3,37] would translate to a greater degree of association between BRIEF-A ratings of working memory and intelligence in adults with WS. Rather, our results indicated that in adults with WS lower IQ score was associated with greater EF deficits only on the BRIEF-A Shift clinical scale. The discrepancy between performance measures of working memory and parent reports of EF and their interrelationships with IQ might reflect a lack of overlap between standardised measures and behavioural ratings of EF within the everyday setting. However, this is unlikely as our results indicate that the Switch condition of the Shape School, a performance-based measure of cognitive flexibility, also showed the strongest association with IQ. Additionally, there is evidence that performance on the Dimensional Change Card Sort or Wisconsin Card Sorting Test, measures of shifting ability, are strongly correlated with IQ in young typically developing children and adolescents [63,64]. Alternatively, there is also debate as to whether aspects of fluid and crystallised intelligence as measured on psychometric intelligence tests accurately reflect underlying constructs of EF [63]. Given the previously reported cognitive variability in WS [50,65,66], more research is needed with larger samples to explore different subgroups to further clarify associations between shifting, working memory, and IQ in adults with WS.

The current study findings have significant clinical implications for a model of EF assessment that situates the adult with a neurodevelopmental disorder within the context of their unique environment. To date, the validity of child and adult rating scales of EF have not been compared for the assessment of EF in adults when mental age and IQ level would be commensurate with the use of a child version. These data confirm that the adult version of the BRIEF is the most valid clinical and research tool for assessment of EF in adults with WS, but assumptions about the validity of rating scales for use in adults should not be premised on findings in typically developing children. Another important clinical implication is the appropriateness of individual items on rating scales of EF for adults with an ID. More research is needed to develop rating scales that capture the profile of EF impairments that are specific to intellectual level in adults, in the same way the Developmental Behaviour Checklist is specific to behavioural and emotional disturbance in children with ID [67].

There are several limitations of the current study that should be acknowledged. Firstly, the small sample size of adults with WS is a limitation in capturing variability in ratings of EF in the everyday setting, as well as the lack of correction for multiple correlations. Equally as limiting as a strict correction for multiple comparisons however, is the risk of Type II errors which are arguably more important to avoid in research into neurodevelopmental disorders [57]. It should also be acknowledged that data reduction techniques such as latent class cluster analysis would have been useful in identifying subtypes of related cases when examining the EF profile in WS. Although the small sample size precluded the application of these data reduction techniques, an analysis of the effect sizes revealed moderate to large effects, suggesting that a larger sample could have provided a better picture of inter-correlations in the EF profile in WS. Future studies should attempt to overcome the issue of small sample size, for example by using international, multi-site studies to explore the profile of everyday EF in adults with WS. Secondly, the BRIEF rating scales may be biased as they relied solely on parent report of everyday EF. Future studies are needed to examine rating scales from multiple informants to validate these findings across different settings. Furthermore, the potential for parent report bias should be acknowledged, as some parents may view the behaviour of their son or daughter in an unreasonably negative (or positive) way. Finally, since previous studies have not considered the impact of comorbid ADHD on EF impairments in individuals with WS [27], it would be beneficial to compare EF profiles of adults with WS with and without a comorbid diagnosis of ADHD, and compare EF profiles in WS with other neurodevelopmental disorders with distinct EF profiles (e.g. Down syndrome, fragile X syndrome, autism).

In summary, the current study is the first to examine the validity of child and adult rating scale measures of EF difficulties in everyday settings in adults with WS. Our results confirm that the BRIEF-A is more sensitive than the BRIEF-C in capturing distinct components of EF within this population. The pattern of associations between performance measures and rating scales are suggestive of a fractionised profile of relative strengths and weaknesses across individual subcomponents of EF in adults with WS. Contrary to previous studies from performance-based assessments [3], the current findings show that shifting was the component of EF most closely associated with IQ in adults with WS. These findings have significant clinical implications for the appropriateness of child measures of EF for use in adults with ID, and for intervention approaches that focus on relative strengths in EF to improve areas of specific weakness in WS.

## Supporting Information

S1 Table The BRIEF-C Rating Scale Structure. (DOCX)

S2 Table The BRIEF-A Rating Scale Structure. (DOCX)

S3 Table Correlations Between the Shape School Test and the BRIEF-C (T Scores). (DOCX)

S4 Table Correlations Between the WJ III COG and the BRIEF-C (T Scores). (DOCX)

S5 Table Correlations Between the Vineland-II and the BRIEF-C (T Scores). (DOCX)

S6 Table Behavioural profile for Adult WS on the Shape School, WJ III COG and Vineland-II. (DOCX)
