# Supplementary material for: Characterising the Profile of Everyday Executive Functioning and Relation to IQ in Adults with Williams Syndrome: Is the BRIEF Adult Version a Valid Rating Scale?
Source: PLoS One. 2015 Sep 10;10(9):e0137628. doi: 10.1371/journal.pone.0137628 (PMC4565670; doi:10.1371/journal.pone.0137628)
Supplement: S1 Table — (DOCX) [file pone.0137628.s001.docx]

*Supplementary Table 1. The BRIEF-C Rating Scale Structure*

| Composite Scores and Indices | Clinical Scales | Behaviour Measured |
| --- | --- | --- |
| **Behaviour Regulation Index (BRI)** | Inhibit | Ability to control behaviour |
|  | Shift | Ability to move from one situation to another |
|  | Emotional Control | Ability to modulate emotional responses |
| **Metacognition Index (MI)** | Initiate | Ability to independently start a task and generate ideas |
|  | Working Memory | Ability to hold information in one’s mind to complete a task |
|  | Plan/Organise | Ability to manage current and future task demands |
|  | Organisation of Materials | Ability to order/organise one’s world and belongings |
|  | Monitor | Ability to evaluate work and behaviour |
| **Global Executive Composite (GEC)** |  | Summary of all clinical scales |

*Note.* BMI = sum of Initiate, Shift, and Emotional Control; MI = sum of Initiate, Working Memory, Plan/Organise, Organisation of Materials, and Monitor; GEC = sum of all clinical scales. Adapted from the “Behavior Rating Inventory of Executive Function” by G. A. Gioia, P. K. Isquith, S. Guy, and L. Kenworthy, 2000a, p. 2. Copyright 2000 by the Psychological Assessment Resources, Inc.
