# Supplementary material for: Characterising the Profile of Everyday Executive Functioning and Relation to IQ in Adults with Williams Syndrome: Is the BRIEF Adult Version a Valid Rating Scale?
Source: PLoS One. 2015 Sep 10;10(9):e0137628. doi: 10.1371/journal.pone.0137628 (PMC4565670; doi:10.1371/journal.pone.0137628)
Supplement: S2 Table — (DOCX) [file pone.0137628.s002.docx]

*Supplementary Table 2. The BRIEF-A Rating Scale Structure*

| Composite Scores  and Indices | Clinical Scales | Behaviour Measured |
| --- | --- | --- |
| **Behaviour Regulation Index (BRI)** | Inhibit | Ability to control behaviour |
|  | Shift | Ability to move from one situation to another |
|  | Emotional Control | Ability to modulate emotional responses |
|  | Self-Monitor | Ability to evaluate one’s own behaviour |
| **Metacognition Index (MI)** | Initiate | Ability to independently start a task and generate ideas |
|  | Working Memory | Ability to hold information in one’s mind to complete a task |
|  | Plan/Organise | Ability to manage current and future task demands |
|  | Organisation of Materials | Ability to order/organize one’s world and belongings |
|  | Task-Monitor | Ability to evaluate one’s own problem-solving |
| **Global Executive Composite (GEC)** |  | Summary of all clinical scales |

*Note.* BMI = sum of Initiate, Shift, Emotional Control, and Self-Monitor; MI = sum of Initiate, Working Memory, Plan/Organise, Organisation of Materials, and Task-Monitor; GEC = sum of all clinical scales. Adapted from “Behaviour Rating Inventory of Executive Function-Adult Version” by R. M. Roth, P. K. Isquith, and G. A. Gioia, G. A., 2005, p. 2. Copyright 2005 by the Psychological Assessment Resources, Inc.
