# Supplementary material for: Characterising the Profile of Everyday Executive Functioning and Relation to IQ in Adults with Williams Syndrome: Is the BRIEF Adult Version a Valid Rating Scale?
Source: PLoS One. 2015 Sep 10;10(9):e0137628. doi: 10.1371/journal.pone.0137628 (PMC4565670; doi:10.1371/journal.pone.0137628)
Supplement: S3 Table — (DOCX) [file pone.0137628.s003.docx]

*Supplementary Table 3. Correlations Between the Shape School Test and the BRIEF-C (T Scores)*

|  | Shape School Conditions | | | | | | | |
| --- | --- | --- | --- | --- | --- | --- | --- | --- |
| BRIEF-C Clinical Scales, Indices, and GEC | Inhibit | |  | Switch | |  | Both | |
|  | (n = 19) | *p* |  | (n = 19) | *p* |  | (n = 19) | *p* |
| **BRI** | **-.07** | **.658** |  | **.00** | **.943** |  | **-.02** | **.875** |
| Inhibit^a^ | .03 | .605 |  | .15 | .322 |  | .02 | .734 |
| Shift^b^ | .05 | .937 |  | -.19 | .229 |  | -.22 | .848 |
| Emotional Control | -.15 | .436 |  | -.02 | .776 |  | .06 | .955 |
| **MI** | **-.27** | **.315** |  | **-.08** | **.955** |  | **-.23** | **.392** |
| Initiate | .02 | .958 |  | .18 | .303 |  | .02 | .764 |
| Working Memory | -.22 | .317 |  | -.09 | .926 |  | -.30 | .310 |
| Plan/Organise | -.30 | .260 |  | -.22 | .490 |  | -.38 | .095 |
| Org. of Materials | -.22 | .319 |  | .16 | .817 |  | .21 | .638 |
| Monitor | -.19 | .503 |  | -.17 | .797 |  | -.26 | .469 |
| **GEC** | **-.19** | **.394** |  | **-.05** | **.945** |  | **-.18** | **.532** |

*Note.* Scores represent Pearson’s correlation coefficient.

^a^ Spearmen’s Rho correlation coefficient between the BRIEF-C Inhibit scale *T* score and the Shape School conditions. ^b^ Spearmen’s Rho correlation coefficient between the BRIEF-C Shift scale *T* score and the Shape School conditions.
