# Supplementary material for: Characterising the Profile of Everyday Executive Functioning and Relation to IQ in Adults with Williams Syndrome: Is the BRIEF Adult Version a Valid Rating Scale?
Source: PLoS One. 2015 Sep 10;10(9):e0137628. doi: 10.1371/journal.pone.0137628 (PMC4565670; doi:10.1371/journal.pone.0137628)
Supplement: S4 Table — (DOCX) [file pone.0137628.s004.docx]

*Supplementary Table 4.Correlations Between the WJ III COG and the BRIEF-C (T Scores)*

|  | WJ III COG Clinical Clusters | | | | | | | |
| --- | --- | --- | --- | --- | --- | --- | --- | --- |
| BRIEF-C Clinical Scales, Indices, and GEC | Working Memory | |  | Broad Attention | |  | Executive Processes | |
|  | (n = 15) | *p* |  | (n = 16) | *p* |  | (n = 15) | *p* |
| **BRI** | **-.01** | **.966** |  | **-.17** | **.499** |  | **-.32** | **.253** |
| Inhibit^a^ | -.04 | .733 |  | -.18 | .176 |  | -.28 | .227 |
| Shift^b^ | -.12 | .329 |  | -.25 | .104 |  | -.46 | .064 |
| Emotional Control | .08 | .799 |  | -.06 | .784 |  | -.19 | .478 |
| **MI** | **.04** | **.824** |  | **-.26** | **.383** |  | **-.21** | **.486** |
| Initiate | .30 | .283 |  | .08 | .849 |  | .11 | .662 |
| Working Memory | -.03 | .958 |  | -.28 | .301 |  | -.26 | .401 |
| Plan/Organise | -.15 | .552 |  | -.37 | .145 |  | -.34 | .184 |
| Org. of Materials | .40 | .147 |  | .22 | .439 |  | .26 | .434 |
| Monitor | -.05 | .888 |  | -.32 | .247 |  | -.31 | .306 |
| **GEC** | **.04** | **.913** |  | **-.22** | **.403** |  | **-.27** | **.350** |

*Note.* Scores represent Pearson’s correlation coefficient.

^a^ Spearmen’s Rho correlation coefficient between the BRIEF-C Inhibit scale *T* score and the WJ III COG clinical clusters. ^b^ Spearmen’s Rho correlation coefficient between the BRIEF-C Shift scale *T* score and the WJ III COG clinical clusters.
