# Supplementary material for: Characterising the Profile of Everyday Executive Functioning and Relation to IQ in Adults with Williams Syndrome: Is the BRIEF Adult Version a Valid Rating Scale?
Source: PLoS One. 2015 Sep 10;10(9):e0137628. doi: 10.1371/journal.pone.0137628 (PMC4565670; doi:10.1371/journal.pone.0137628)
Supplement: S5 Table — (DOCX) [file pone.0137628.s005.docx]

*Supplementary Table 5. Correlations Between the Vineland-II and the BRIEF-C (T Scores)*

|  | Vineland-II Domains | | | | | | | | | | | | | | |
| --- | --- | --- | --- | --- | --- | --- | --- | --- | --- | --- | --- | --- | --- | --- | --- |
|  |  |  |  |  |  |  | Maladaptive Behaviour Subscales | | | | | | | |  |
| BRIEF-C Clinical Scales, Indices, and GEC | Adaptive Behaviour Composite | |  | Socialisation Domain | |  | Maladaptive Behaviour Index | |  | Internalising Behaviours | |  | Externalising Behaviours | | |
|  | (n = 16) | *p* |  | (n = 16) | *p* |  | (n = 15) | *p* |  | (n = 15) | *p* |  | (n = 15) | *p* | |
| **BRI** | **.15** | **.632** |  | **.22** | **.402** |  | **-.18** | **.718** |  | **-.16** | **.614** |  | **.11** | **.407** | |
| Inhibit^a^ | .22 | .573 |  | .26 | .361 |  | -.32 | .314 |  | -.26 | .535 |  | -.07 | .787 | |
| Shift^b^ | -.27 | .397 |  | -.18 | .738 |  | -.27 | .403 |  | -.17 | .542 |  | -.22 | .741 | |
| Emotional Control | .32 | .341 |  | .35 | .241 |  | .04 | .724 |  | -.01 | .962 |  | .44 | .048* | |
| **MI** | **-.13** | **.833** |  | **-.10** | **.944** |  | **.13** | **.889** |  | **-.13** | **.453** |  | **.23** | **.694** | |
| Initiate | -.10 | .909 |  | -.13 | .876 |  | .04 | .752 |  | -.06 | .751 |  | .10 | .559 | |
| Working Memory | -.09 | .950 |  | -.13 | .913 |  | -.09 | .803 |  | -.30 | .197 |  | -.11 | .803 | |
| Plan/Organise | -.18 | .757 |  | -.17 | .806 |  | .22 | .712 |  | -.01 | .750 |  | .31 | .591 | |
| Org. of Materials | .13 | .968 |  | .20 | .820 |  | -.01 | .949 |  | -.15 | .699 |  | .06 | .715 | |
| Monitor | -.22 | .747 |  | -.12 | .840 |  | .11 | .960 |  | -.08 | .532 |  | .23 | .644 | |
| **GEC** | **-.03** | **.939** |  | **.00** | **.748** |  | **.03** | **.944** |  | **-.17** | **.476** |  | **.15** | **.532** | |

*Note.* Scores represent Pearson’s correlation coefficient.

^a^ Spearmen’s Rho correlation coefficient between the BRIEF-C Inhibit scale *T* score and the Vineland-II domains. ^b^ Spearmen’s Rho correlation coefficient between the BRIEF-C Shift scale *T* score and the Vineland-II domains.

* *p* < 0.05.
