# Supplementary material for: Characterising the Profile of Everyday Executive Functioning and Relation to IQ in Adults with Williams Syndrome: Is the BRIEF Adult Version a Valid Rating Scale?
Source: PLoS One. 2015 Sep 10;10(9):e0137628. doi: 10.1371/journal.pone.0137628 (PMC4565670; doi:10.1371/journal.pone.0137628)
Supplement: S6 Table — (DOCX) [file pone.0137628.s006.docx]

*Supplementary Table 6. Behavioural profile for Adult WS on the Shape School, WJ III COG and Vineland-II*

|  | Shape School Conditions | | |  | WJ III COG Clinical Clusters | | |  | Vineland-II Domains | | | | |
| --- | --- | --- | --- | --- | --- | --- | --- | --- | --- | --- | --- | --- | --- |
|  |  |  |  |  |  |  |  |  |  |  | Maladaptive Behaviour Subscales | | |
|  | Inhibit  (n = 19) | Switch  (n = 19) | Both  (n = 19) |  | Working Memory  (n = 15) | Broad Attention  (n = 16) | Executive Processes  (n = 15) |  | Adaptive Behaviour Composite  (n = 16) | Socialisation Domain  (n = 16) | Maladaptive Behaviour Index  (n = 15) | Internalising Behaviours  (n = 15) | Externalising Behaviours  (n = 15) |
| *M* | 1.09 (.00-2.50) | .40 (.00-1.07) | .43 (.00-1.36) |  | 63.41 (21-89) | 51.33 (3-80) | 58.76 (23-94) |  | 51.39 (27-84) | 59.83 (33-83) | 18.41 (16-23) | 19.12 (16-23) | 16.88 (14-21) |
| *SD* | .53 | .27 | .28 |  | 18.79 | 24.10 | 18.06 |  | 14.39 | 11.76 | 1.91 | 2.15 | 2.00 |
| %^a^ | - | - | - |  | 53% | 78% | 71% |  | 89% | 89% | - | - | - |

*Note.* ^a^The percentage of adult WS participants who obtained a clinically significant score of 69 or below for the WJ III COG and the Vineland-II Adaptive Behaviour Composite and Socialisation Domain are provided. No clinically significant ranges are available for the Vineland-II Maladaptive Behaviour Scales and The Shape School conditions. The minimum and maximum scores of WS participants from the Shape School, WJ III COG and Vineland-II are in parentheses.
